# Some Annual Meetings

**Published:** 1918-04-20

**Authors:** 


### SOME ANNUAL MEETINGS.

### Charing Cross Hospital.

A Unique Position.

A remarkable speech in connection with Charing Cross Hospital was made by Mr. George Verity, J.P., its Chairman, at the annual general Court of Governors held last week. In moving the adoption of the report for 1917, he said that were it not for the anxious times through which we were passing the meeting of Governors would be an occasion for sounding the clarions and waving the banners of joy; for since the hospital was founded, just one hundred years ago, by that great lover of mankind, Dr. Benjamin Golding, it had never been in such a favourable position as it was at the present moment. Tracing the history of the institution from its small beginnings to its painful and struggling adolescence, he went on to speak of the time of its prime, when new buildings were put up, extensions made, and the Nurses' Home was built. But, alas! (he continued) those extensions meant running into a dreadful debt, including a mortgage of £85,000 and a building debt of over £20,000. But that was not all. Its income decreased, and parts of both the old and the new buildings had to be closed owing to lack of funds. However, as the result of steady and continuous work, special benevolence on the part of the public, shoulder to shoulder loyalty and self-denial, he was at last in a position to announce that the mortgage had been swept away, that the builder's debt was a thing of the past, and that the closed wards and the unequipped theatres were things of memory.

The hospital to-day was carrying on its work as efficiently as it was humanly possible, free and unencumbered by debt, and equipped on the latest scientific lines. Further, it showed nearly the most economical working of any similar institution in London, and stood extremely high in the eyes of the medical profession. Princess Louise, its President, by her munificence and thoughtful enthusiasm, had buoyed them up and brought them into the present lagoon of happy and peaceful contentment. The only shadow over their heads was the stir in Government and journalistic circles to destroy more or less voluntary institutions and substitute those founded on the "hard principles of the tax-gatherer." He hoped that no hurried decision would be come to, and that there would be no interference with what was the most glorious appanage of the British public—its charitable institutions. With regard to the military wards, he was happy to say that the authorities had found no hospital more easy to get on with, and none more satisfactory in its results. Sir Thomas Dewar, speaking of the Chairman's strenuous work on behalf of the hospital, described him as a hotbed of genius and a whirlwind of energy.

### Royal National Orthopædic Hospital.

In the absence of Colonel the Earl of Denbigh, Chairman of the hospital, Mr. Edmund Dean, the Deputy-Chairman, presided at the annual Court of Governors held last week at Great Portland Street, W. It was reported that during the past year 950 in-patients and 5,961 out-patients, whose attendances had amounted to 41,349, had been cared for. The figures showed a marked increase on previous years. Expenditure had increased from £17,059 in 1916 to £20,135 for the year under review. Contrary to the experience of nearly all similar institutions, the sum derived from legacies had increased. At the joint request of the Army Council and the Ministry of Pensions the Committee of Management had undertaken since the beginning of the year the treatment of discharged disabled soldiers in place of serving soldiers, and 150 beds, instead of 170, which was the number at the disposal of military patients last year, had been allocated for that purpose. Thus a further twenty beds had been set free for the use of civilians. Soldiers to the number of 3,774 had been cared for since the beginning of the war, and a workshop for their use was being erected on the vacant site adjoining the Nurses' Home.

